# Don’t sweat the small stuff: skin mechanisms of sodium homeostasis and associations with long-term blood pressure

**DOI:** 10.1042/CS20230163

**Published:** 2023-05-18

**Authors:** Joshua S. Speed, David M. Pollock, John S. Clemmer

**Affiliations:** 1 Department of Physiology and Biophysics, University of Mississippi Medical Center, Jackson, MS 39216, U.S.A.; 2 Division of Nephrology, Department of Medicine, University of Alabama at Birmingham, Birmingham, U.K.

**Keywords:** renal physiology, skin, sodium homeostasis, sweat

## Abstract

Despite the overwhelming evidence that the kidney is the principal regulator of chronic blood pressure though the ability to sense pressure and adjust blood volume accordingly, recent clinical and preclinical evidence suggests that skin clearance of Na^+^ through sweat significantly contributes to long-term blood pressure and risk of hypertension. Evidence indicates that changes in skin Na^+^ content negatively associate with renal function, and factors that influence the concentration of Na^+^ in sweat are affected by major regulators of Na^+^ excretion by the kidney such as angiotensin and aldosterone. In addition, known regulatory mechanisms that regulate the amount of sweat produced do not include changes in Na^+^ intake or blood volume. Because of these reasons, it will be hard to quantify the contribution of Na^+^ clearance through sweat to blood pressure regulation and hypertension. While Chen et al. demonstrate significant negative associations between sweat Na^+^ concentration and blood pressure, it is likely that Na^+^ clearance through the skin has a short-term influence on blood pressure and sweat Na^+^ concentration is most likely a biomarker of renal function and its key role in hypertension.

Although a relatively small part of the body of his work, Arthur Guyton is frequently known for his widely accepted theory that the kidney regulates long-term blood pressure by its ability to increase or decrease extracellular fluid and blood volume through excretory function. His hypotheses most likely took root in the late 1950s or early 1960s when his group demonstrated that epinephrine infusion increases urine output when infused at a rate that acutely increased blood pressure through peripheral vasoconstriction; however, at higher infusion rates, renal adrenergic receptors were activated and urine output decreased even though a marked elevation in blood pressure was observed [1]. Over the next couple of decades, it became clear that long-term blood pressure was regulated by the kidneys' ability to excrete Na^+^, the major osmolyte in extracellular fluid, as water typically follows to maintain stable plasma concentrations of its components [2]. Once this physiological principle was established, many factors were discovered that can influence blood pressure’s relationship with renal excretory function by affecting renal hemodynamics or directly affecting Na^+^ transport along the renal tubules. Some of these factors include the renin–angiotensin–aldosterone system, sympathetic nervous system, natriuretic peptides, nitric oxide, and endothelin. The ability of human bodies to suppress or activate these systems in coordination with hydration status and Na^+^ intake makes them critical buffers that maintain constant blood pressure in the face of constantly changing Na^+^ and water intake. Equally important are the circadian rhythms observed in a number of these systems that lead to reduced Na^+^ and water excretion while we sleep. Therefore, it is important to understand short term factors/buffers that influence renal excretory function to influence blood pressure versus the long-term ability of the kidney to control blood pressure.

Growing clinical and preclinical evidence that the skin clearance of Na^+^ contributes to long-term blood pressure has contradicted the idea that the kidney is the sole regulator of chronic blood pressure. Over the last decade, it has been recognized that the skin can sequester Na^+^, most of which is stored non-osmotically. Investigators have sought to determine the different mechanisms that control Na^+^ storage in the skin. One prevailing theory is when salt intake is elevated, increased extracellular tonicity leads to infiltration of macrophages that release vascular endothelial growth factor c to promote lymph vessel formation in the skin through which stored Na^+^ can be cleared back into circulation [3]. Interestingly, blocking any part of this pathway leads to salt-sensitive hypertension [3]. Controversy over Guyton's renal-body fluid feedback mechanism in the control of blood pressure was fueled by the association between skin Na^+^ and hypertension coupled with work by Conn and Louis indicating that aldosterone reduces Na^+^ concentration in sweat [4] and the negative association observed between blood pressure and sweat Na^+^ concentration [5]. However, the initial experiments that demonstrated the importance of skin Na^+^ stores and the ability to clear Na^+^ from the circulation were performed in rodents, which lack sweat glands or any other mechanism to clear water or electrolytes through the skin, essentially making the kidney the only pathway for ultimate clearance of excess Na^+^ in rodents. Humans on the other hand, can lose appreciable amounts of Na^+^ through the skin through sweat, and therefore, understanding these mechanisms could have an impact on management of hypertension.

The study by Chen et al. appears to indicate that the ability of the body to alter Na^+^ concentration in sweat is a *causative* mechanism to regulate blood pressure and contribute to hypertension [6]. However, as discussed by Chen et al., mechanisms that influence renal excretory function and skin Na^+^ content and sweat concentration largely overlap and may simply represent an *association*. For example, in the present study, patients on angiotensin-converting enzyme inhibitors (ACEs) or angiotensin receptor blockers (ARBs) have higher concentration of Na^+^ in the sweat compared with those not taking ACEs or ARBs [6]. Also, glomerular filtration rate negatively correlates with skin Na^+^ concentration [7], and conditions in which renal function is reduced, such as chronic kidney disease or heart failure, lead to increased skin Na^+^ content compared to healthy subjects, while treatments that increase renal Na^+^ excretion such as diuretics reduce skin Na^+^ content in these patients as shown through Na^+^ MRI [8]. In addition, patients with Conn’s syndrome have reduced Na^+^ concentration in the skin, while patients treated with aldosterone inhibitors have the opposite [9]. The associations between mechanisms related to skin Na^+^ content and blood pressure would at face value make it easy to speculate that skin Na^+^ loss could be an important regulator of extracellular Na^+^ and water balance. The major issue with this statement is that Na^+^ stored in the skin can only be cleared through lymph vessels, and ultimately by the kidney, or through sweat, which is produced in response to increased body temperature, not by changes in Na^+^ intake. For example, acute exposure to high environmental heat increases sweat volume and Na^+^ loss through sweat, leading to reduced intravascular volume. Renal compensation results in reduced sodium excretion via increased angiotensin II and aldosterone to mitigate any further decreases in plasma volume [9]. Repeated days of heat exposure in previously non-acclimatized people leads to an aldosterone-mediated reduction in sweat Na^+^ concentration and less Na^+^ loss through sweat as compared with the initial days of heat exposure. Na^+^ balance is achieved in the face of elevated aldosterone due to Na^+^ ‘escape’ through the kidney [9]. Thus, the ability of angiotensin and aldosterone to reduce sweat Na^+^ concentration appears to be an important mechanism to mitigate Na^+^ loss in individuals that have large daily rates of sweat, such as outdoor workers in warm climates, which, if anything, is associated with hypotension. Taken together, it is most likely that sweat Na^+^ concentration is a product of volume status and renal excretory function and not an important regulator of long-term blood pressure control.

Guyton's idea of chronic blood pressure regulation by the kidney that is taught throughout medical schools today indicates that renal output of Na^+^ will always equal intake minus a negligible non-renal loss through feces and sweat to maintain Na^+^ and fluid homeostasis [10]. This mechanism works over days and weeks, where other short-term buffer mechanisms can impact blood pressure throughout the course of a single day. An ideal buffer for Na^+^ homeostasis would have to be precisely regulated in the face of changes in Na^+^ intake. For instance, every known mechanism that influences renal excretory function can be activated or suppressed as Na^+^ intake changes. In addition, circadian rhythms in blood pressure and Na^+^ excretion are observed in normal physiology, where blood pressure dips and Na^+^ excretion is reduced during the inactive period. However, in rodents, there is no evidence that skin Na^+^ content changes throughout the course of a day. Our lab, to our knowledge, is the only one to report skin Na^+^ content over a 24-h period in rodents. Our results show no difference in skin Na^+^ or water content in a normotensive rat over a 4-h period, whether rats were on a normal or high NaCl diet. Interestingly, rats that lacked endothelin type B receptors were associated with reduced renal excretory function and developed a clear diurnal pattern in skin Na^+^ that was mirrored by water and coupled with severe salt-sensitive hypertension characterized by exaggeration of diurnal blood pressure rhythms [11]. As noted, rats do not have the ability to sweat, so deducing conclusions on whether the skin can influence long-term blood pressure control by increasing Na^+^ clearance through sweat is not feasible through rodent studies. A study using Na-MRI to get an idea of diurnal patterns of Na^+^ content in skin and sweat would greatly improve our understanding of diurnal mechanisms of Na^+^ balance and quantify the potential Na^+^ buffering capacity of the skin.

From the current study by Chen et al., it is clear that sweat Na^+^ content can be modified by several of the same mechanisms that influence renal Na^+^ excretion [12]. What is less clear is the contribution of this Na^+^ loss to blood pressure regulation and whether mechanisms that reduce Na^+^ loss through sweat would contribute to hypertension independent of renal function (Figure 1). Given that sweat is controlled by body heat and not Na^+^ intake, it is more likely that these mechanisms have short-term influence on blood pressure and that sweat Na^+^ concentration could serve as a biomarker of renal homeostatic mechanisms at play. More chronic intervention studies using the techniques of Chen et al. are warranted in humans to properly understand the contribution of Na^+^ loss through sweat to blood pressure regulation.

**Figure 1:**
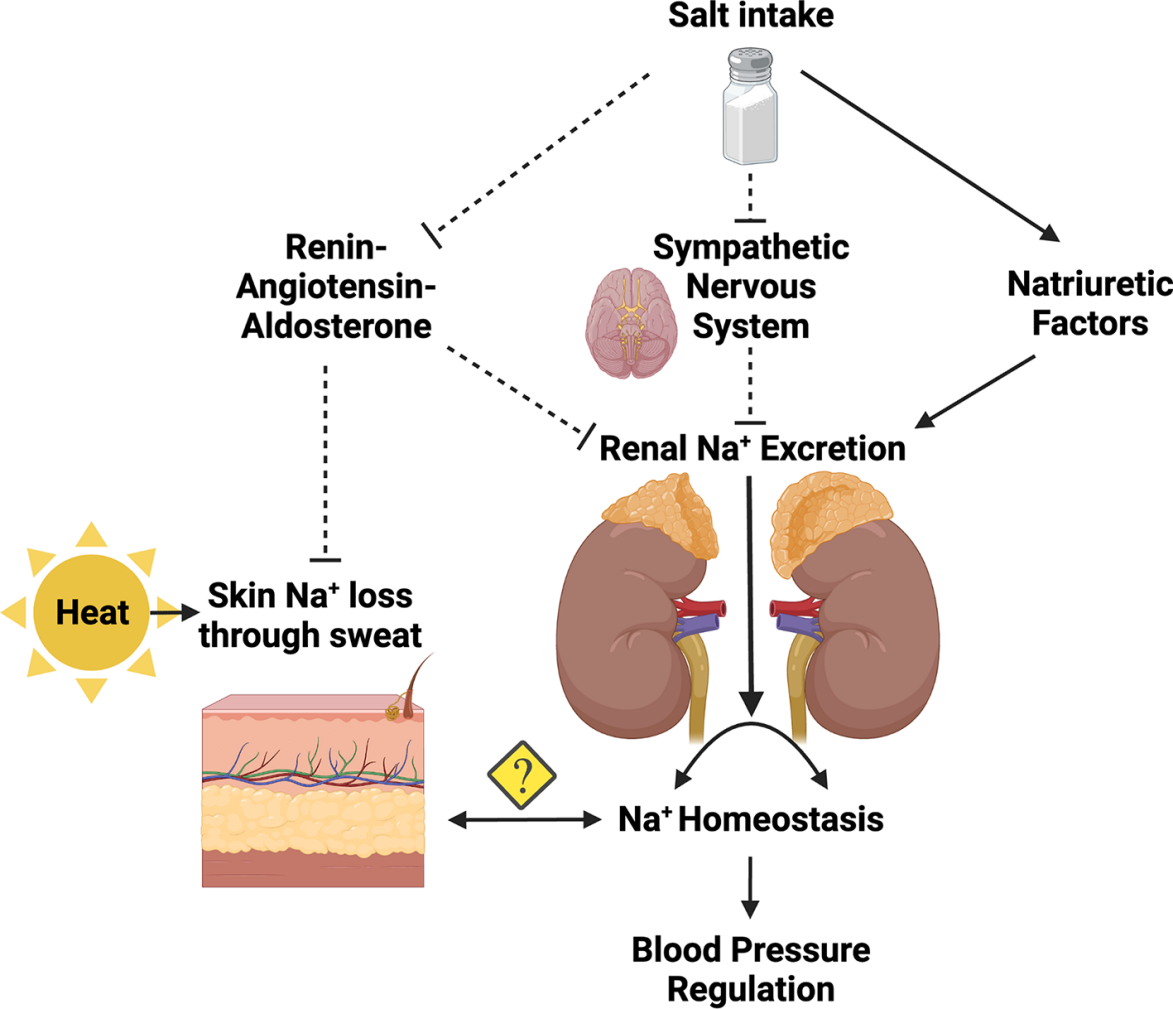
Schematic integrating mechanisms of Na^+^ homeostasis and blood pressure regulation Solid lines with arrows represent positive input while dashed, capped lines represent negative input.

## Data Availability

There are no data included in this commentary.

## Open Access

Open access for this article was enabled by the participation of University of Mississippi in an all-inclusive *Read & Publish* agreement with Portland Press and the Biochemical Society under a transformative agreement with Individual.

## Abbreviations

ACE: angiotensin-converting enzyme inhibitor
ARB: angiotensin receptor blocker
